# Non‐Bonded Interaction Driven Morphology Evolution of CuMOF@MXene Energetic Composites: Synergistic Optimization of Thermal Stability and Combustion Performance

**DOI:** 10.1002/advs.202508755

**Published:** 2025-07-13

**Authors:** Ke‐Juan Meng, Xinwen Ma, Kunyu Xiong, Xiaoxia Ma, Iftikhar Hussain, Momang Tian, Kaili Zhang

**Affiliations:** ^1^ Department of Mechanical Engineering City University of Hong Kong 83 Tat Chee Avenue Hong Kong China; ^2^ State Key Laboratory of Explosion Science and Safety Protection Beijing Institute of Technology Beijing 100081 China

**Keywords:** combustion, energetic metal‐organic frameworks, non‐bonded interaction, thermal stability, Ti_3_C_2_T*_x_* MXene

## Abstract

Hybrid materials with tunable properties, particularly metal‐organic frameworks (MOFs)@MXene composites have emerged as a cutting‐edge research focus different applications. In this study, a novel energetic CuMOF composed of Cu^2+^ and 3‐(tetrazol‐5‐yl) triazole is synthesized, followed by the development of CuMOF@MXene composites (CuMOF@MX*x*) via a facile one‐step hydrothermal method. This approach leverages the interfacial “Ti─O···Cu” non‐bonded interactions to achieve composites with diverse morphologies. Subsequently, both experiments and theoretical calculations verify the presence of these non‐bonded interactions and their influence on morphology, thermal property, and combustion performance of the composites. The morphologies of composites transition from a flower‐like structure to a spherical shape with the addition of Ti_3_C_2_T*_x_* MXene. Furthermore, simultaneous thermogravimetry‐differential scanning calorimetry tests, along with non‐isothermal kinetic analysis, indicate that CuMOF@MX_4_ has a higher initial temperature of 354.2 °C for runaway reaction and a larger activation energy of 211.58 kJ mol^−1^, compared to CuMOF (314.9 °C and 202.56 kJ mol^−1^). In addition, the combustion tests demonstrate that CuMOF@MX*x* exhibit more intense and rapid combustion. This work demonstrates the critical role of the non‐bonded interaction between CuMOF and Ti_3_C_2_T*_x_* interfaces in regulating the structure and properties of the composites, and the possible mechanism of this process is also elucidated.

## Graphical abstract

This work presents a strategy for preparing energetic composites based on energetic metal‐organic frameworks and MXene. This approach leverages the interfacial “Ti─O···Cu” non‐bonded interactions to achieve energetic composites with diverse morphologies and performance. The study further elucidates the relation between structure and performance. Overall, this work presents a new paradigm for the design of energetic composite.

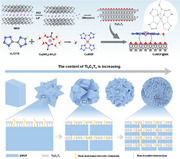

## 1 Introduction

Over the past few decades, energetic metal‐organic frameworks (EMOFs) composed of nitrogen‐rich energetic ligands and transition metal have gradually emerged as a promising topic and have gained significant attentions in the field of energetic materials.^[^ ^1^ ^]^ Benefiting from their tunable structure, high density, elevated nitrogen content and excellent thermal stability, EMOFs are considered as a new generation high‐energy materials for civilian and military purposes, particularly in primary/secondary explosives, biocidal agents, combustion catalysts, and pyrotechnics additive.^[^ ^2^ ^]^ On one hand, the utilization of high‐energy bonds of nitrogen‐rich ligands, such as C─N, N─N, N═N and N≡N (with average bond energies of 273, 160, 418 and 954 kJ mol^−1^, respectively), provides a significant advantage in increasing the energy output.^[^ ^3^ ^]^ The breaking of these bonds during decomposition and combustion releases substantial energy, thereby improving the overall energy performance of EMOFs.^[^ ^4^ ^]^ One the other hand, the diversity of nitrogen‐rich ligands, including pyrazole, tetrazole and triazole, provides multiple coordination sites for commonly used transition metal ions, facilitating the formation of 1D, 2D, and 3D structures based on coordination chemistry.^[^ ^5^ ^]^ Among these, 3D coordination geometries can provide superior structural reinforcement for the energetic groups, making the selection of suitable nitrogen‐rich ligands critical for constructing thermal stability 3D structures that enhance the safety in application and transportation.

3‐(Tetrazol‐5‐yl) triazole (H_2_Tztr) exemplifies a nitrogen‐rich ligand that combines the high nitrogen content of tetrazole ligands with the stability of triazole ligands, achieving a nitrogen content of 71.54%. The presence of numerous nitrogen atoms with lone‐pair electrons on its heterocyclic structure allows for versatile coordination modes with metal ions, facilitating the multiple dimensional expansion required to develop thermally stable and high energetic EMOFs.^[^ ^6^ ^]^ However, the intrinsically low thermal conductivity and microscale size of EMOFs and the limited diffusion of combustion products still restrict the further improvement in the combustion propagation and energy release efficiency.^[^ ^7^ ^]^

To further boost the thermal stability and combustion propagation, researchers have incorporated classical 2D materials such as graphene oxide (GO) into EMOFs.^[^ ^8^ ^]^ However, MXene (M*_n_*_+1_X*_n_* T*_x_*), an emerging class of 2D transition‐metal carbides, nitrides, and carbonitrides, remain largely unexplored for morphology modulation and performance enhancement of energetic materials.^[^ ^9^ ^]^ MXenes are characterized by their unique layered structure, where M represents an early transition metals (Ti, V, Mo), X denotes carbon and/or nitrogen atoms, and T_x_ corresponds to surface terminations (O, OH, F) generated during the etching process.^[^ ^10^ ^]^ With a high specific surface area that facilitates superior interfacial interactions,^[^ ^11^ ^]^ high electrical conductivity (>5000 S cm^−1^),^[^ ^12^ ^]^ exceptional thermal transport properties,^[^ ^13^ ^]^ and controlled surface chemistry through functional group engineering,^[^ ^14^ ^]^ MXene offer promising opportunities as additives for novel energetic composites. These properties can potentially address critical challenges in optimizing energy density, improving combustion efficiency, and enhancing material stability through strategic surface functionalization and nanoarchitecture design. More specifically, the layered structure of MXenes can serve as efficient channels for heat and mass transfer, thereby accelerating energy propagation and regulating combustion pathways. In addition, the abundant functional groups on their surface can engage in various interactions (e.g., hydrogen bonding, van der Waals forces) with EMOFs, leading to the formation of tight interface contacts and synergistically optimizing both energy output and composite stability.^[^ ^15^ ^]^

In this study, we first synthesized energetic coordination polymers, CuMOF, based on H_2_Tztr and transition metal copper ions, and analyzed their crystal structures and performance characteristics. Subsequently, we developed a strategic interfacial engineering approach where functional groups of Ti_3_C_2_T*_x_* nanosheets selectively interact with unsaturated metal sites in CuMOF through non‐bonded interactions, achieved through a one‐pot solvothermal method. In the resulting CuMOF@MXene composites (CuMOF@MX*x*), the surface functional groups of Ti_3_C_2_T*_x_* not only inhibit the further growth of CuMOF but also enhance interfacial interaction.

The evolution of morphology and structure, thermal performance and combustion performance, were systematically characterized. Furthermore, the mechanisms underlying structural evolution and performance enhancement were investigated and discussed. This study not only presents a new paradigm for the design of energetic composites based on EMOFs and MXenes but also expands the theoretical understanding of non‐bonded interaction between EMOFs and MXene.

## 2 Results and Discussion

### 2.1 Single‐Crystal X‐Ray Diffraction of CuMOF

**Figure 1:**
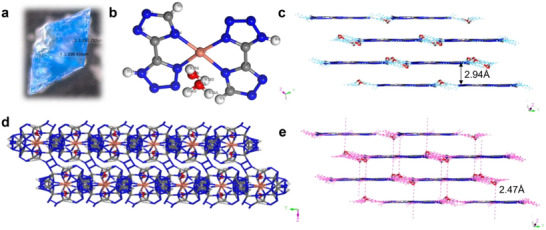
a) The single crystal of CuMOF. b) The asymmetric unit of CuMOF. c) The hydrogen‐bonding in 3D supramolecular framework. d) The 2D planar layer in CuMOF. e) The van der Waals interactions in CuMOF.

Single‐crystal X‐ray diffraction analysis of CuMOF was conducted using a Bruker D8 VENTURE TXS PHOTON II equipped with a Mo Kα radiation (λ ¼ 0.71073 Å) employing ω and ψ scan modes. The single‐crystal structure was solved by direct methods using SHELX‐2019/3.^[^ ^16^ ^]^ The results elucidated that the blue crystal (**Figure** **1**a), acquired through the gradual evaporation of the reaction mixture at ambient temperature, crystallized within the monoclinic space group *C*12/*c*1, with a crystal density of 1.882 g cm^−3^ at 296 K.

As illustrated in Figure 1b, the asymmetric unit consists of one Cu(II) ion, two H_2_Tztr ligands, and two coordinated water molecules, resulting in the empirical formula Cu(H_2_Tztr)_2_(H_2_O)_2_. The Cu(II) ion adopts a distorted octahedral coordination environment (Figure S1, Supporting Information), coordinated by four nitrogen atoms from two H_2_Tztr ligands (Cu─N(1) = 1.996 Å, Cu─N(7) = 2.086 Å, Cu─N(8) = 2.006 Å. Cu─N(14) = 1.939 Å). The H_2_Tztr ligand adopted bidentate chelating modes within the CuMOF framework. The mononuclear units extended into a 3D supramolecular framework via hydrogen‐bonding (Figure 1c) between molecules in the same layer and van der Waals interactions (Figure 1e) between molecules in distinct layers. In CuMOF, the O(1A), O(1B), N(4) and N(11) atoms acted as hydrogen‐bonding donors, interacting with the acceptors at the N(10‐13), N(3), N(4), O(1A) and O(1B) atoms of the adjacent molecule. The distances for these interaction were as follows: 1.829 Å for O(1A)─H(1AA)···N(10) and O(1A)─H(1AB)···N(10), 2.152 Å for O(1A)─H(1AA)···N(11), 2.143 Å for O(1A)─H(1AB)···N(4) and N(11)─H(11)···O(1A), 2.080 Å for O(1B)─H(1BB)···N(10), 2.382 Å for O(1B)─H(1BA)···N(12), 1.339 Å for O(1B)─H(1BA)···N(13), 1.988 Å for N(4)─H(4)···O(1A) and 2.335 Å for N(4)─H(4)···O(1B). Other details of the crystal data, data collection parameters and refinement statistics of CuMOF are given in Table S1 (Supporting Information). This intricate network of hydrogen bonds and van der Waals interactions underscores the structural integrity and stability of the CuMOF framework, highlighting its potential applications in energetic materials.

### 2.2 Structural Features of CuMOF and CuMOF@MX*x* Composites

**Figure 2:**
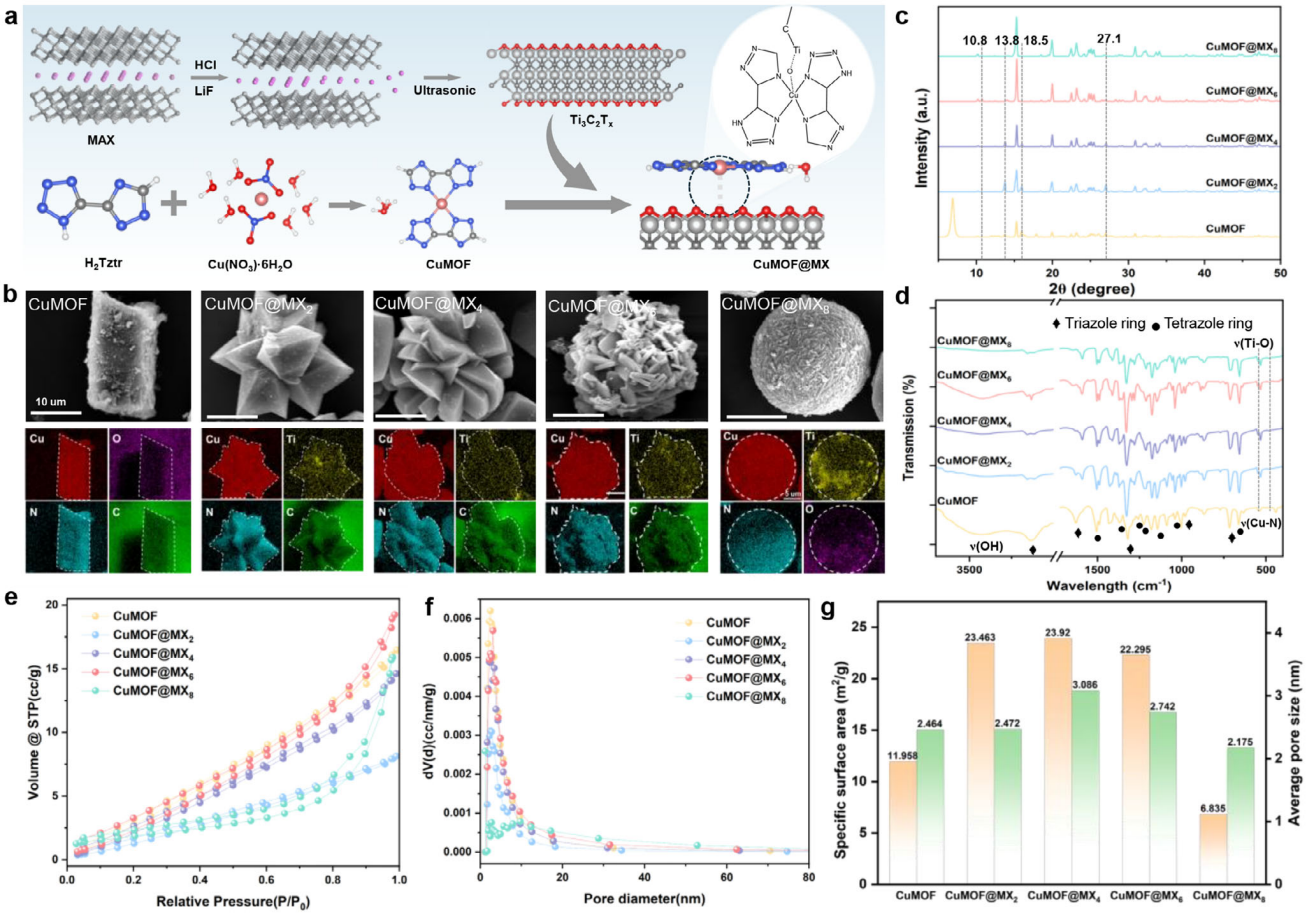
Synthesis and characterization of CuMOF and CuMOF@MX*_x_* composites. a) Schematic illustration of the synthesis process. (b) SEM images and elemental dispersion mappings of Cu, O, N, C and Ti in CuMOF, CuMOF@MX_2_, CuMOF@MX_4_, CuMOF@MX_6_, and CuMOF@MX_8_ composites. Structural comparison and analysis of CuMOF and CuMOF@MX*_x_*. c) PXRD patterns. d) FT‐IR spectra. e) N_2_ adsorption−desorption isotherm. f) Pore size plots. g) Specific surface area and average pore size of CuMOF and CuMOF@MX*_x_* composites.

The microstructure, morphology and elemental composition of CuMOF and CuMOF@MX*_x_* composites, where *x* represents the mass percentage of Ti_3_C_2_T*_x_* incorporated via the solvothermal method (**Figure** **2**a), were investigated using scanning electron microscopy (SEM, FEI Quanta 450) coupled with energy dispersive spectroscopy (EDS). As illustrated in Figure 2b, CuMOF exhibited a 3D prismatic structure, corroborating the findings obtained through optical microscopy. EDS mapping of CuMOF revealed a relatively uniform distribution of Cu (red), N (blue), C (green), and O (purple), with element compositions of 3.0%, 42.5%, 44.9% and 9.6%, respectively. In the case of the CuMOF@MX*x* composites, a significant presence of evenly distributed Ti element was observed on the surface of CuMOF, forming a tightly interconnected heterogeneous flower‐/spherical‐like structure. The EDS spectra of the selected region confirmed the presence of Cu, C and N, along with Ti (yellow), with Cu uniformly distributed throughout CuMOF@MX*x* composite. This uniform distribution suggests that the modification of CuMOF with Ti_3_C_2_T*_x_* was successfully achieved. Furthermore, when the doping amount of Ti_3_C_2_T*_x_* was 8wt%, a noticeable transition of the 3D structure from flower‐like to spherical occurs, accompanied by a significant increase in the Ti element content on the surface, as shown in Figure S2 (Supporting Information). This morphological transformation may be attributed to the enhanced interaction between CuMOF and Ti_3_C_2_T*_x_*, which facilitates the formation of a more compact and integrated composite structure.

The chemical composition was investigated by the Fourier transform infrared spectroscopy (FTIR, Thermo Fisher Scientific Nicolet iN10, America), X‐ray powder diffractometer (XRD, Rigaku SmartLab) and X‐ray photoelectron spectroscopy (XPS, Thermo Scientific Nexsa, America). The Brunauer–Emmett–Teller (BET) surface area and pore size distribution data were attained by using the Quantachrome Autosorb NOVA 2200e tests at 77.3 K by N_2_ adsorption/desorption. The XRD patterns (Figure 2c) indicated that all prepared samples (CuMOF and CuMOF@MX*x*) exhibited good crystallinity, as evidence by sharp and intense diffraction peaks. Notably, several prominent peaks located at 15.3°, 19.9°, 23.1°, 27.1°, and 30.9° of CuMOF persisted, with their intensities increasing upon the incorporation of Ti_3_C_2_T*_x_* sheets. However, the strongest peak of CuMOF at 6.8°disappeared, which may be attributed to the influence of Ti_3_C_2_T*_x_* on the growth direction of the CuMOF crystals. Additionally, new characteristic peaks at 10.8° and 18.5°, representing the (002) crystal surface of Ti_3_C_2_T*_x_*, were observed in the series of CuMOF@MX*x* composites.^[^ ^17^ ^]^ Furthermore, as the content of Ti_3_C_2_T*_x_* content increased, the intensity of the diffraction peaks of CuMOF@MX at 13.8° and 27.1° initially intensified, subsequently diminished, and eventually vanished. This trend confirms that Ti_3_C_2_T*_x_* content plays a critical role in modulating crystal growth along these directions.

The FT‐IR spectra of the CuMOF and CuMOF@MX*x* composites are shown in Figure 2d. In general, the intense and broad band at approximately 3200–3700 cm^−1^ could be related to the O─H bonds and hydrogen‐bonded of H_2_O molecules, implying the presence of water molecules in CuMOF.^[^ ^18^ ^]^ More detailly, the peaks of CuMOF at 1512 cm^−1^, 1368 cm^−1^, 1258 cm^−1^,1226 cm^−1^, 1147 cm^−1^, 1040 cm^−1^, and 662 cm^−1^ attributed to the characteristic vibrations of the tetrazole ring skeleton, whereas the infrared absorption bands at 3132 cm^−1^, 1628 cm^−1^, 1327 cm^−1^, 979 cm^−1^, and 716 cm^−1^ were ascribed to the characteristic vibrations triazole ring.^[^ ^19^ ^]^ Notably, two peaks at 1002 cm^−1^ and 478 cm^−1^ probably matched the Cu─N stretching vibration, indicating coordination bonding.^[^ ^20^ ^]^ During the synthesis process of CuMOF@MX*x* composites, Cu ions were initially anchored onto Ti_3_C_2_T*_x_* through electrostatic interactions, serving as nucleation sites for in situ growth of CuMOF. Therefore, the peak for the vibration of Cu─N at 1002 cm^−1^ of CuMOF@MX*x* became significantly weaker than in bare CuMOF, which was due to weakening of the Cu─N coordination bond by the interaction between Cu and Ti_3_C_2_T*_x_*. Additionally, a new absorption peak emerged at 525 cm^−1^ in the spectra of CuMOF@MX*x*, with its intensity increasing alongside Ti_3_C_2_T*_x_* content. This peak can be assigned to the Ti─O groups from Ti_3_C_2_T*_x_*,^[^ ^21^ ^]^ further confirming that Ti_3_C_2_T*_x_* restricts CuMOF growth by interacting with Cu^2+^ ions.

Figure 2e illustrates the N_2_ adsorption and desorption isotherms of CuMOF and CuMOF@MX*x* composites. The N_2_ adsorption desorption isotherms of CuMOF and CuMOF@MX*x* could be classified as type‐IV with a type‐H3 hysteresis loop, indicating the presence of mesoporous structures (i.e., pore sizes between 2 and 50 nm).^[^ ^22^ ^]^ The BET specific surface areas of CuMOF, CuMOF@MX_2_, CuMOF@MX_4_, CuMOF@MX_6_ and CuMOF@MX_8_ were calculated to be 11.958, 23.463, 23.920, 22.295, and 6.835 m^2^ g^−1^, respectively. Notably, CuMOF@MX_4_ exhibited a high specific surface area (around 2 times that of CuMOF), which might belong to its special flower‐like structure. The corresponding pore size distribution plots were shown in Figure 2f. The average pore sizes of CuMOF, CuMOF@MX_2_, CuMOF@MX_4_, CuMOF@MX_6_ and CuMOF@MX_8_ were about 2.464, 2.472, 3.086, 2.742 and 2.175 nm (Figure 2g), respectively. CuMOF@MX_8_ exhibited a lower pore size and surface area than CuMOF, representing a more compact structure, which is consistent with SEM observations. The results showed that CuMOF@MX_4_ possessed a high specific surface area and mesoporous structure, and these pore sizes provide channels for electron transfer during its thermal decomposition, thereby enhancing its thermal decomposition performance.

**Figure 3:**
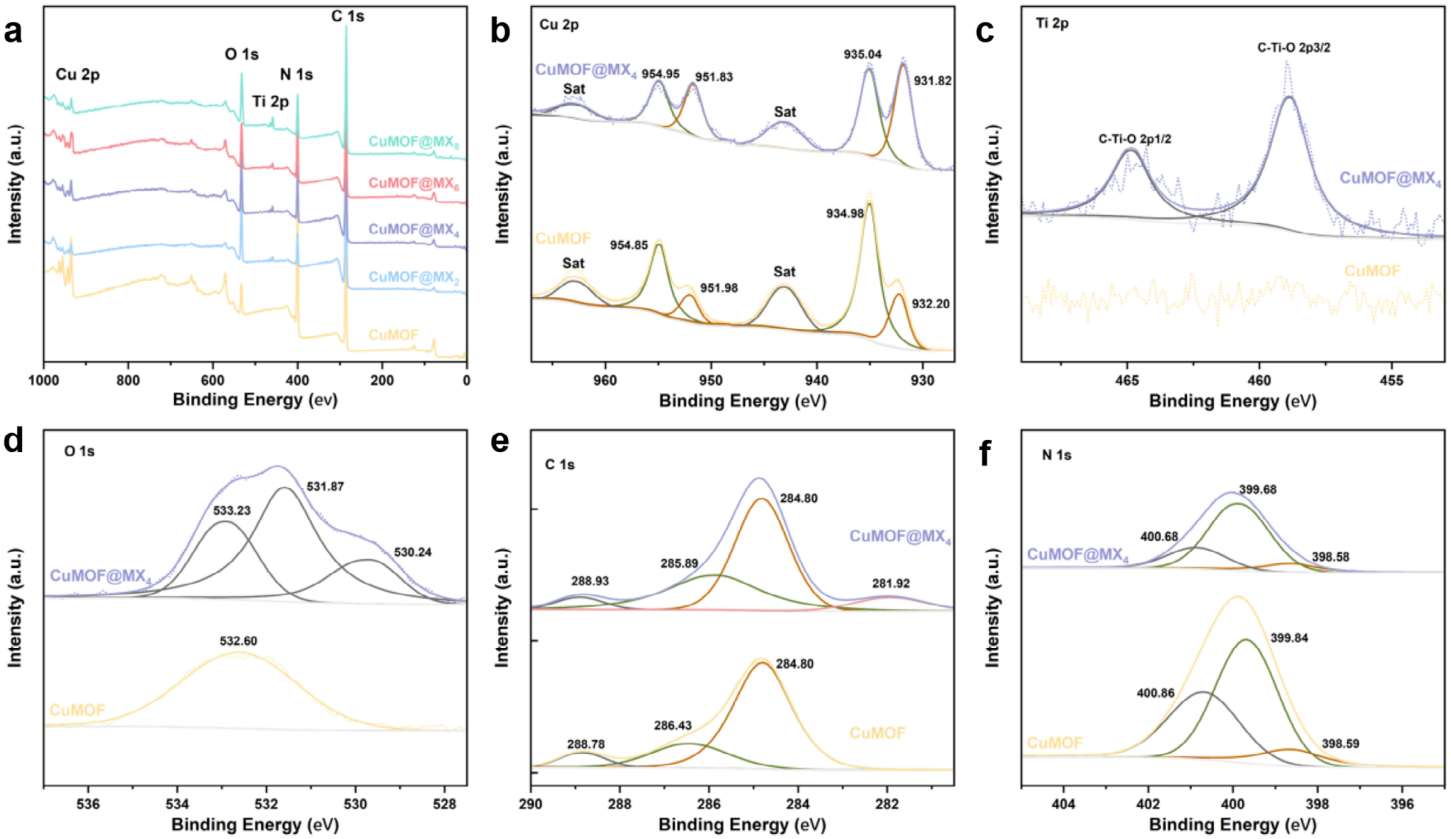
XPS spectra of CuMOF and CuMOF@MX*_x_* composites. a) XPS survey spectra. The high‐resolution b) Cu 2p, c) Ti 2p, d) O 1s, e) C 1s and f) N 1s XPS spectra of CuMOF and CuMOF@MX_4_.

To further determine chemical composition and valence states, X‐ray photoelectron spectroscopy (XPS) analyses were performed on both CuMOF and CuMOF@MX*x* composites. The XPS survey spectra (**Figure** **3**a) confirmed the presence of Cu, N, C, and O in CuMOF. Meanwhile, the signals of Ti 2p, Cu 2p, O 1s, N 1s, and C 1s, located at 459.24, 934.93, 532.95, 400.14, and 285.99 eV, were observed in the XPS full spectra of CuMOF@MX*x* composites. Figure 3b illustrates that the Cu 2p spectra for both CuMOF and CuMOF@MX*x* composites exhibited Cu 2p_1/2_ and Cu 2p_3/2_ peaks,^[^ ^23^ ^]^ two satellite peaks^[^ ^24^ ^]^ and a double peak (932.2/931.82 eV and 951.98/951.83 eV), demonstrating the existence of Cu^2+^ and Cu^+^ in the composites.^[^ ^25^ ^]^ Notably, the double peak in CuMOF@MX_4_ moved to lower binding energies with increased intensity compared to those in CuMOF, which could be attributed to the interaction between the Cu^+^ and surface functional groups (─O, ─OH, etc.) from Ti_3_C_2_T*_x_* nanosheets.

In Figure 3c, the Ti 2p spectra of CuMOF@MX_4_ were resolved into Ti 2p_1/2_ (464.8 eV) and Ti 2p_3/2_ (458.9 eV) peaks, indicating an abundance of active Ti^4+^ sites were formed during solvothermal reactions,^[^ ^26^ ^]^ and they played an important role in the in situ anchoring of CuMOF via the “Ti─O···Cu” interaction.^[^ ^27^ ^]^ Further, three peaks located at 533.23, 531.87, and 530.24 eV appeared in the O 1s spectra of CuMOF@MX_4_ (Figure 3d), which could be attributed to the H_2_O,^[^ ^28^ ^]^ Ti─(OH)*_x_*,^[^ ^29^ ^]^ and Cu─OH,^[^ ^30^ ^]^ indicating that CuMOF and Ti_3_C_2_T*_x_* were linked by the “Ti─O···Cu” interaction. In Figure 3e, the C 1s peaks of CuMOF observed at 284.8, 286.43, and 288.78 eV could be assigned to the C─C, C─N, and C═N─C correspond to the ligands. An additional peak appeared at 281.92 eV of CuMOF@MX_4_ corresponded to C─Ti bond of Ti_3_C_2_T*_x_*.^[^ ^31^ ^]^ As illustrated in Figure 3f, the N 1s spectrum of CuMOF had three peaks at 398.59, 399.84, and 400.86 eV, which were assigned to the N═N─N, N─Cu, and N─N═C, respectively. Overall, the XPS results ensured that the “Ti─O···Cu” interaction forms during the in situ growth of CuMOF via anchoring at active Ti sites on the surface of Ti_3_C_2_T*_x_*.

### 2.3 Thermal Stability and Nonisothermal Kinetic Analysis

**Figure 4:**
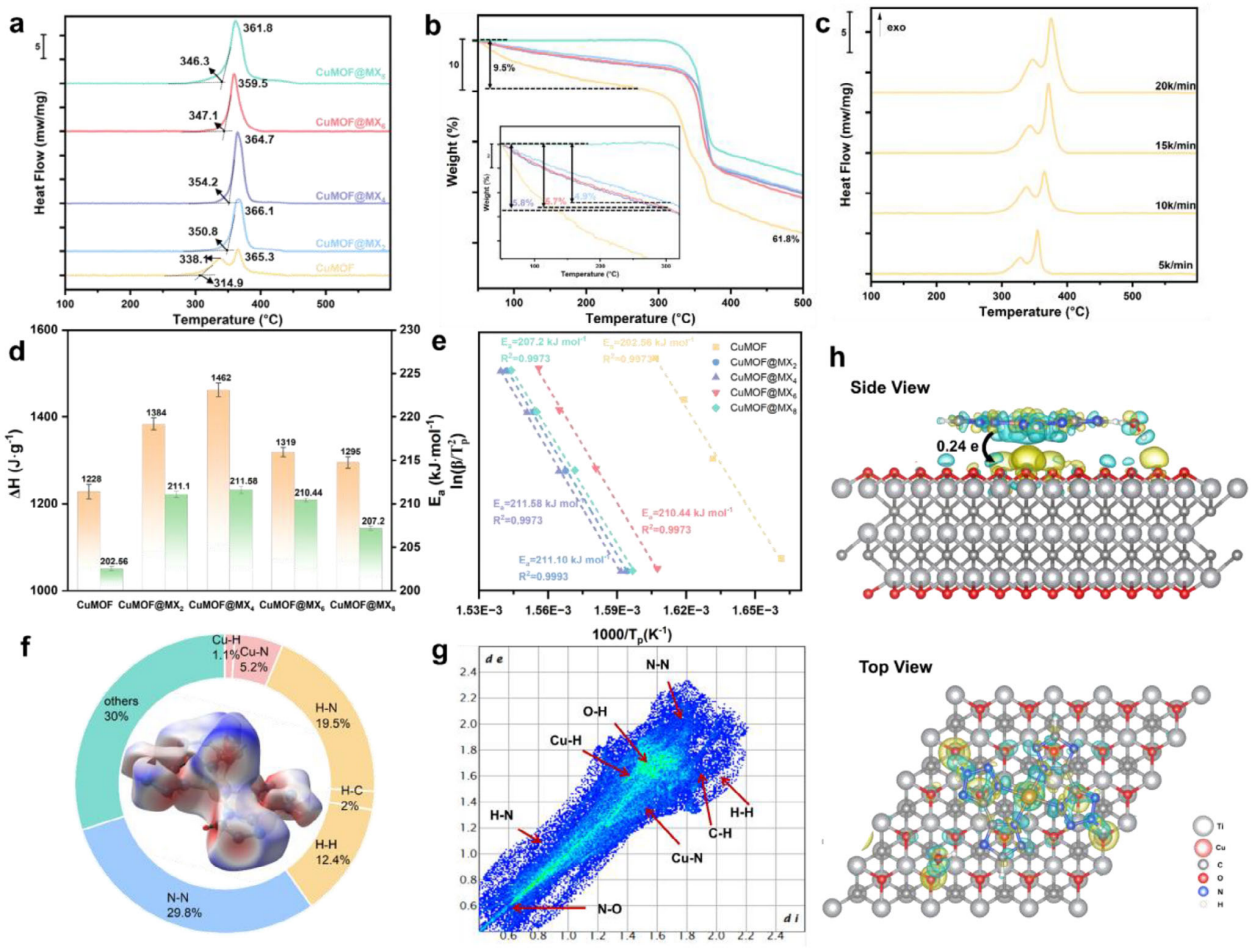
Thermal stability and non‐isothermal kinetic analysis of CuMOF and CuMOF@MX*x* composites. a) DSC results at 10 °C min^−1^. b) TG plots. c) DSC curves of CuMOF at different heating rates of 5, 10, 15, and 20 °C min^−1^. d) Heat release and apparent activation energy. e) Kissinger curves. f) Hirshfeld surfaces of Cu‐MOF. g) 2D fingerprints of Cu‐MOF. h) The side view and top view of calculation results.

Thermal analysis was performed on simultaneous thermogravimetry‐differential scanning calorimetry instrument (TG‐DSC, METTLER TGA/DSC 3+) under an argon atmosphere in the temperature range of 50–600 °C. As illustrated in **Figure** **4**a and Table S2 (Supporting Information), as‐prepared materials exhibited higher thermal stability compared to other EMOFs,^[^ ^32^ ^]^ attributed to their rigid ligand structure, bidentate chelating coordination mode, and layer‐by‐layer crystal packing.

The initial temperature of runaway reaction (T_i_) of CuMOF is 314.9 °C, followed by two consecutive thermal decomposition peaks with peak temperatures of 338.1 and 365.3 °C, respectively. These two stages correspond to the breaking of ligand bonds and ring‐opening dissociation of the tetrazole in its ligand, which produces gases such as N_2_ and CO_2_, respectively.^[^ ^33^ ^]^ In contrast, only one strong exothermic peak was observed for all CuMOF@MX*x* samples, representing breaking of the coordination bond is delayed and completed in a single step with the ring‐opening dissociation of ligand. The initial temperature of runaway reaction of CuMOF@MX_2_, CuMOF@MX_4_, CuMOF@MX_6_ and CuMOF@MX_8_ was 350.8 °C, 354.2 °C, 348.1 °C and 346.3 °C, respectively. They were 31.4–39.3 °C higher than CuMOF, which may arise from interaction between Ti_3_C_2_T*_x_* and MOF. In addition, the heat release could be obtained by integrating the shadow area into the DSC curves. It is worth noting that the total heat release of all CuMOF@MX*x* samples showed an increase trend as shown in Figure 4d. Specifically, CuMOF@MX_4_ has the largest heat output, reaching 1462 J g^−1^, which is approximately 1.2 times that of CuMOF. However, when the Ti_3_C_2_T*_x_* content increases to 6%, the heat output begins to decrease because the heat release mainly arises from the breaking of the tetrazole ring in the ligand. The increase of Ti_3_C_2_T*_x_* content could inevitably lead to the decrease of CuMOF content, that is, the decrease of the content of tetrazole rings, thus reducing the heat release.

The TG curve showed that CuMOF undergoes three mass loss stages as shown in Figure 4b. The first mass loss stage began heating up to 300 °C, accompanied by about 9.5% mass loss, which was attributed to the loss of two coordinated water molecules (8.1% theoretically), and the extremely small heat release of the coordinated water molecule was not seen in the DSC curve. The second and third weight loss processes were observed at around 300–346.5 °C and 346.5–382.2 °C and are considered to be the collapse of the main framework. The mass percentage of residue in the final product is 61.8%, which was higher than the calculated value of 38% for the ideal condensed phase product with CuO and C as shown in Equation 1. This is due to the fact that CuMOF is oxygen‐lean and decomposes incompletely in an inert atmosphere, resulting in hydrocarbons in the final product. The weight loss of the first stage is 4.9%, 5.8% and 5.7% for CuMOF@MX_2_, CuMOF@MX_4_, CuMOF@MX_6_, respectively, which was lower than that of CuMOF. In particular, it is only 0.4% for CuMOF@MX_8_, which may be due to the formation of interactions between the O groups on the Ti_3_C_2_T*_x_* surface and the Cu^2+^ in CuMOF, which reduces the probability of crystalline H_2_O coordination in the lattice.

(1) $$\mathrm{Cu}C_{6}H_{8}N_{14}O_{2}\to \mathrm{CuO}+7N_{2}+\mathrm{CO}+5C+4H_{2}$$

The thermal decomposition behavior of as‐prepared samples was further investigated at ramping rates of 5, 10, 15, and 20 °C min^−1^ for the kinetics study. The detailed data are shown in Table S3 (Supporting Information). With the increase in heating rate, the exothermic peak area enlarged visibly, and the peak temperature increased significantly as shown in Figure 4c and Figure S3 (Supporting Information). The thermal decomposition kinetics were investigated by calculating the activation energies of the samples by a Kissinger equation^[^ ^34^ ^]^ as presented in Equation 2 as follows:

(2) $$\ln\;\left(\frac{\beta }{T_{p}^{2}}\right)=\;\ln\left(-\frac{AR}{E_{a}}f^{\prime }\left(\alpha_{p}\right)\right)-\frac{E}{RT_{p}}$$

where 𝛽 is the linear heating rate in (K min^−1^); *T*_p_ and α_p_ denote the decomposition peak temperature (K) and reaction progress at the maximum peak temperature, *A* and *R* represent the pre‐exponential factor and the ideal gas constant (8.314 J mol^−1^ K^−1^), respectively, *E*_a_ is the apparent activation energy, kJ mol^−1^. According to Equation (2), the term ln (𝛽/$T_{p}^{2}$) varies linearly with 1/*T*_p_ as shown in Figure 4e, the activation energy and pre‐exponential factor of sample thermal decomposition can be separately obtained from the slope and intercept of the straight line. As illustrated in Figure 4d, the apparent activation energy of CuMOF@MX_2_, CuMOF@MX_4_, CuMOF@MX_6_ and CuMOF@MX_8_ (211.10, 211.58, 210.44 and 207.20 kJ mol^−1^) was slightly higher than CuMOF (202.56 kJ mol^−1^ of CuMOF). This revealed that the CuMOF@MX*x* had better thermal stability under the rapid heating process compared to CuMOF.

To gain a deeper understanding of the correlations between the properties and intermolecular interactions of CuMOF, comprehensive analyses involving Hirshfeld surfaces (Figure 4f) and 2D fingerprint spectra (Figure 4g) were analyzed by Crystalexploere21.5 software, which is a powerful tool for the investigation of intermolecular interaction.^[^ ^35^ ^]^ It can provide a quantitative analysis of the intermolecular interactions of a compound through fingerprint plots.^[^ ^36^ ^]^ As shown in Figure 4f, atomic molecules were in contact with each other closer than the sum of their van der Waals radii was highlighted in red on the dnorm​ surface. The longer contacts were blue, while the contacts around the sum of the van der Waals radii were white. In CuMOF, the red surface similarly denotes strong close‐contact interactions due to predominant hydrogen bonding interactions. The Cu exhibits robust Cu─N(5.2%) and Cu─H(1.1) interactions, suggesting the formation of coordination bonds between Cu and the N atoms in the ligand. Furthermore, intermolecular hydrogen bonding arising from O─H, C─H, H─H and N─H interactions amount to 34%, thereby providing bolstered stability to the framework. This comprehensive analysis demonstrates the role of intermolecular interactions in improving the stability of CuMOF, suggesting its potential application as a more stable energetic material.

The inherent reasons behind the improvement of thermal performance were investigated through density functional theory (DFT) to calculate the binding energy and interactions of electrons between CuMOF and Ti_3_C_2_T*_x_*. Detailed information about the simulation method was shown in Supporting Information. In this study, Bader charge analysis was employed to quantitatively assess the charge transfer in the CuMOF@MX*x*. The results indicated that the energies of CuMOF, Ti_3_C_2_T*_x_*, and CuMOF@MX*x* were ‐197.1511, ‐1623.0752, and ‐1822.2016 eV, respectively. Given that the binding energy (*E*_b_) between CuMOF and Ti_3_C_2_T*_x_* serves as an indicator of thermodynamic stability, the *E*_b_ of CuMOF@MX*x* was calculated using Equation 3. It was determined to be ‐1.9753 eV, which suggests the presence of stable interactions between CuMOF and Ti_3_C_2_T*_x_*, such as hydrogen bonding, ionic bonding, or van der Waals forces, favoring the formation of stable composites. Differential charge density analysis was carried out for the charge transfer direction, which led to a clearer understanding of the charge redistribution between Ti_3_C_2_T*_x_* and CuMOF and revealed the charge transfer characteristics of the CuMOF@MX*x* composite system. As illustrated in Figure 4h, there was a significant interaction and charge redistribution between Ti_3_C_2_T*_x_* and CuMOF, with the yellow and cyan regions representing the accumulation and dissipation of charge densities, respectively. Consequently, combined with Bader charge analysis, it was confirmed that this charge redistribution resulted in the transfer of 0.24 e of electrons per unit cell from CuMOF to the O‐active sites of Ti_3_C_2_T*_x_*, establishing electronic interactions between these components. Furthermore, it is noted that the distance between Cu and O was measured at 2.765 Å, which deviated from the conventional Cu─O distance of 1.8–2.2 Å.^[^ ^37^ ^]^ This suggests the presence of weak interactions between CuMOF and Ti_3_C_2_T*_x_*, such as weak coordination forces or van der Waals forces, rather than the formation of a chemical bond. Consequently, the combination of DFT calculations demonstrated that the CuMOF@MX*x* has stable structure, thereby exhibiting enhanced kinetics and thermal stability.

(3) $$\;E_{b}=E_{\mathrm{total}}\;-E_{A}-E_{B}$$

The *E*_total_, *E*_A_ and *E*_B_ represent the energy of compound structure, Ti_3_C_2_T*_x_* and CuMOF in the compound structure, respectively.

### 2.4 Combustion Performance

**Figure 5:**
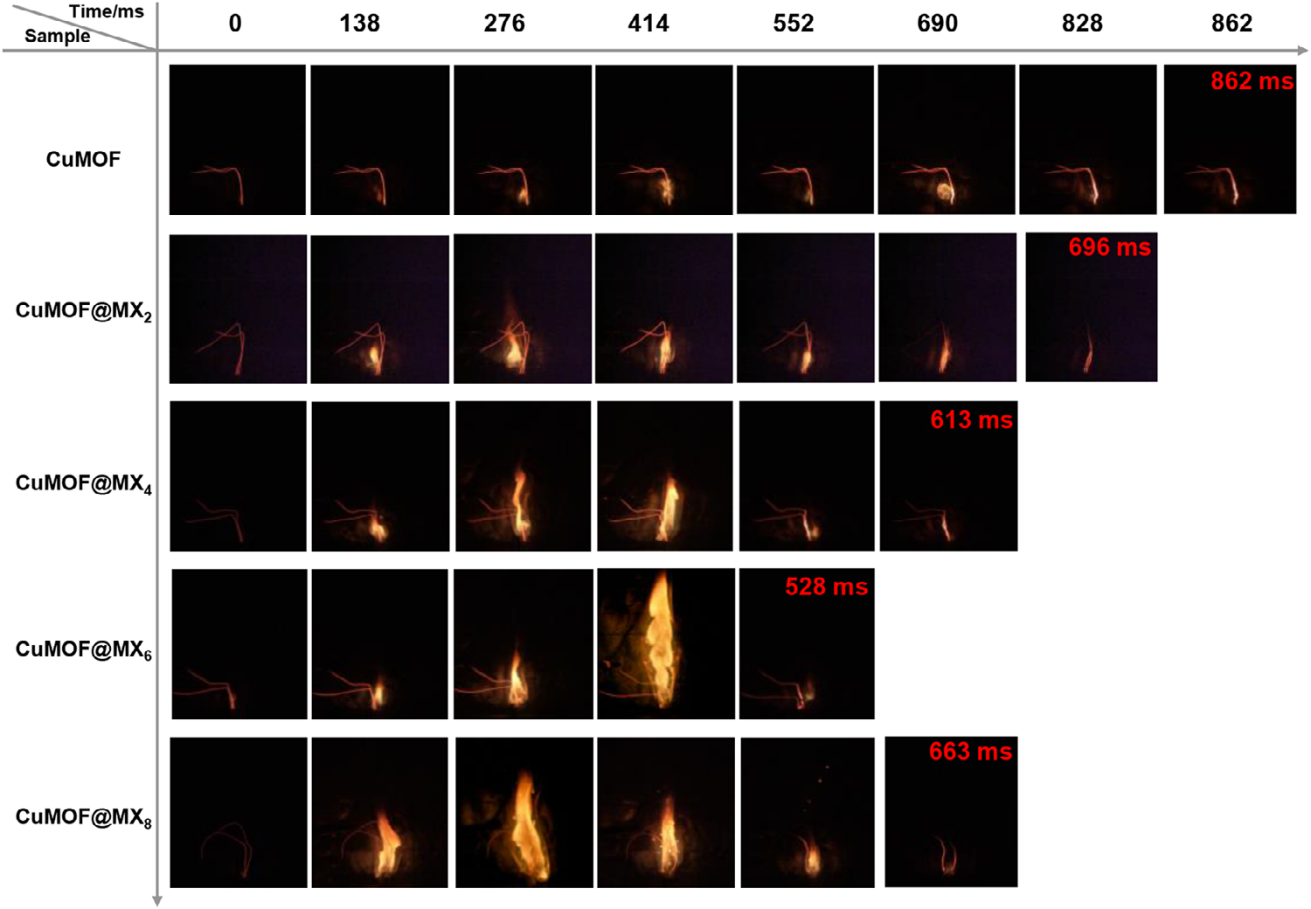
Burning sequences of CuMOF and CuMOF@MX*x* composites.

Combustion experiments of CuMOF and CuMOF@MX*x* were conducted under an air atmosphere by using the homemade test equipment as shown in Figure S4 (Supporting Information). High‐speed camera was used to capture sequential images of ≈10 mg loose powder samples loaded into alumina crucibles. The exposure time and frame rate were kept consistent for all captured images. The combustion performance of CuMOF@MX*x* is better than that of pure CuMOF from the burning sequence obtained from combustion experiments (**Figure** **5**), showing larger maximum flame area and rapid combustion. To be specifically, due to the intrinsic low thermal conductivity, micro‐size of CuMOF and the large amount of gas produced by thermal decomposition to quickly push away the unreactive sample, forming a physical barrier between the hot spot and remaining sample, resulting in low combustion propagation and energy release efficiency, which is manifested as long combustion time (862 ms) and less obvious flame (1.99 cm^2^).^[^ ^7^ ^]^ In contrast, the incorporation of Ti_3_C_2_T*_x_* improved the combustion performance of the composites, as evidenced by a significant reduction in combustion time and enhancement of flame area of CuMOF@MX*x*. When the content of Ti_3_C_2_T*_x_* was 2%, 4%, 6% and 8%, the combustion time was reduced by 19.3%, 28.9%, 38.7% and 23.1%, respectively. At the same time, the maximum flame area is enhanced by 52.7%, 133.2%, 549.2% and 400.5% as shown in Figure S5 (Supporting Information). When the content of Ti_3_C_2_T*_x_* is 8%, the combustion performance begins to decrease, which may be due to the decrease in the effective content of energetic materials, resulting in a decrease in the energy performance of the composites.

### 2.5 Mechanisms Analysis

**Figure 6:**
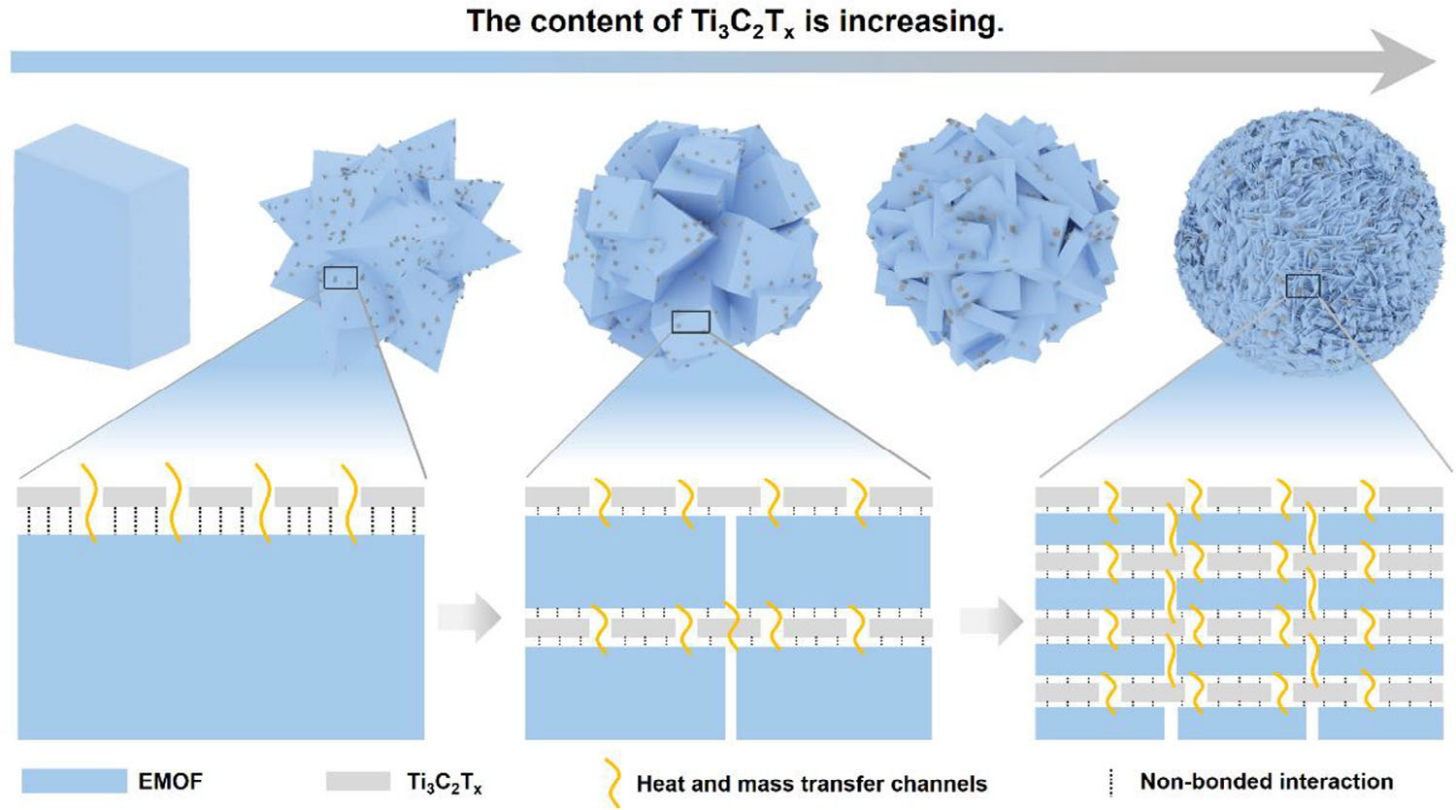
Mechanisms for Ti_3_C_2_T*_x_* in regulating structure, thermal and combustion performance.

As illustrated in **Figure** **6**, based on the results and discussion regarding the roles of Ti_3_C_2_T*_x_* in the morphology evolution and performance control of CuMOF@MX*x*, we propose possible mechanisms for Ti_3_C_2_T*_x_* in regulating structure, thermal and combustion properties.

First, the initial incorporation of Ti_3_C_2_T*_x_* into CuMOF induced non‐bonded interfacial interactions between the components, as evidenced by theoretical calculations and physicochemical characterization. This interaction effectively inhibited crystal overgrowth, triggering a morphological transition from cubic geometry to flower‐like architectures with surface‐anchored Ti_3_C_2_T*_x_* nanosheets. Progressive Ti_3_C_2_T*_x_* loading (8 wt%) ultimately yielded tightly packed spherical structures through oriented attachment mechanisms.

Furthermore, the structure–property relationship analysis revealed significant thermal stability variations. Controlled Ti_3_C_2_T*_x_* addition (4 wt%) enhanced thermal decomposition resistance, manifested by 4.5% increase in apparent activation energy (*E*_a_) and 26.6 °C elevation in thermal decomposition temperature (*T*_d_), attributable to the non–bonded interaction between Cu^2+^ of CuMOF and O atom of Ti_3_C_2_T*_x_*. However, excessive Ti_3_C_2_T*_x_* incorporation (>4 wt%) caused reduction in crystal size and decreased effective CuMOF content, resulting in reduction of *E*_a_ and thermal stability due to compromised structural integrity.

Last, the integration of Ti_3_C_2_T*_x_* into the composites significantly enhances combustion performance through three synergistic mechanisms. First, nanosheets serve as effective crystal growth inhibitors, constraining the development of single‐crystalline CuMOF domains. This size confinement effect results in reduced particle dimensions, which facilitates rapid ignition of CuMOF@MX*x*. Second, the heterointerface between Ti_3_C_2_T*_x_* and CuMOF generates interconnected microchannels. These hierarchical architectures promote efficient heat transfer and accelerated mass diffusion during the exothermic process.^[^ ^38^ ^]^ Third, the incorporated Ti_3_C_2_T*_x_* may provide dual catalytic enhancement. On the one hand, Ti or TiO_2_ caused by combustion may demonstrate catalytic activity for CuMOF combustion. On the other hand, surface functional groups (─O, ─OH) optimize the oxygen balance, creating a self‐sustaining oxidative microenvironment that promotes complete combustion.^[^ ^39^ ^]^ However, the combustion performance was reduced when the Ti_3_C_2_T*_x_* addition reached 8 wt%, which was mainly due to the reduction of the effective content of the energetic material (CuMOF).

## 3 Conclusion

In summary, we synthesized a new CuMOF (Cu(H_2_Tztr)_2_(H_2_O)_2_), based on which we presented a strategy to successfully fabricate CuMOF@MX*x* composites with high thermal stability and enhanced combustion performance. The CuMOF@MXx composites were constructed via the “Ti─O···Cu” non‐bonded interaction between 2D Ti_3_C_2_T*_x_* MXene and CuMOF to form a composite with mesoporous structure, in which the non‐bonded interaction promotes the thermal stability of the composites, and the mesoporous structure provides the channel for the combustion to improve the heat and mass transfer. Excitingly, both the experiments (XRD, FTIR and XPS) and the theoretical simulation results further demonstrate the existence of the non‐bonded interaction and the successful preparation of CuMOF@MX*x* composites. With the integration of 4wt% Ti_3_C_2_T*_x_*, the CuMOF@MX_4_ could achieve good thermal properties, with an initial temperature of 354.2 °C for runaway reaction and an activation energy of 211.58 kJ mol^−1^, which exceeds that of CuMOF at 314.9 °C and 202.56 kJ mol^−1^. In addition, CuMOF@MX_6_ was determined to possess the best combustion performance due to its short combustion time (528 ms) and large maximum flame area (12.92 cm^2^). This proof‐of‐concept work provides theoretical basis for the application of 2D MXene in the field of energetic materials to achieve high thermal stability and combustion performance.

## 4 Experimental Section

Caution! H_2_Tztr, CuMOF and CuMOF@MX*x* are energetic materials, explosions of which may occur in certain conditions. Appropriate safety precautions should be taken when preparing and handling, especially when the composites are prepared on a large scale.

### General

3‐(1H‐tetrazol‐5‐yl)‐1H‐triazole (H_2_Tztr) and Mxene (Ti_3_C_2_T*_x_*) used in the experiment were purchased from Jinan Henghua Sci. & Tec. Co., Ltd and Jiangsu XFNANO Materials Tech. Co., Ltd, respectively. Deionized water was obtained from MilliQ + purification system (18 MΩ cm). ACS‐grade concentrated ethanol absolute was purchased from Anaqua Global International Inc., Ltd (Hong Kong). Other reagents were purchased from Shanghai Aladdin Biochemical Technology Co., Ltd without further purification.

### Synthesis of CuMOF

First, 67.5 mg (0.5 mmol) H_2_Tztr was dissolved in deionized water (30 mL) at 70 °C, then 120.8 mg (0.5 mmol) copper nitrate hexahydrate (Cu(NO_3_)_2_·6H_2_O, purity > 99.5%) was added at room temperature. The reaction was stirred vigorously at 60 °C until a homogeneous solution was obtained. The solution was then poured into a kettle for followed hydrothermal reaction. It was heated to 120 °C at a rate of 10 °C h^−1^, and then the mixture was reacted for 60 h. After that, it was slowly cooled to room temperature at a rate of 10 °C h^−1^. A blue powdered product (CuMOF) was obtained after centrifuging, washing, and drying. The blue single crystals were obtained by slow evaporation crystallization from solution.

### Synthesis of CuMOF@MXx Composites

Initially, a Ti_3_C_2_T*_x_* solution with a concentration of 0.5 mg mL^−1^ was prepared using ultrasonic treatment for 60 min. The specific preparation process for the hybrid material is exemplified by CuMOF@MX_2_, where “2” denotes the mass percentage of Ti_3_C_2_T*_x_* added. The preparation processes for other composites are consistent, with variations only in the volume of the Ti_3_C_2_T_x_ solution added, while the volume of deionized water is adjusted to ensure a total solution volume of 30 mL. First, 67.5 mg (0.5 mmol) H_2_Tztr was dissolved in deionized water (22.3 mL) at 70 °C. Subsequently, 7.7 mL Ti_3_C_2_T*_x_* solution, containing 3.85 mg of Ti_3_C_2_T*_x_*, was added dropwise while stirring at 70 °C for 1 h. After that, 120.8 mg (0.5 mmol) Cu(NO_3_)_2_·6H_2_O was added, and the mixture was stirred for 1 h. The resulting solution was then transferred to a 50 mL stainless steel autoclave for hydrothermal reaction under the following conditions: the temperature was increased to 120 °C at a rate of 10 °C h^−1^ and maintained for 60 h, followed by cooling to room temperature at a rate of 10 °C h^−1^. The powdered samples were obtained after centrifugation, washing, and drying.

CCDC 2425082 contains the supplementary crystallographic data for this paper. These data can be obtained free of charge from The Cambridge Crystallographic Data Centre via https://www.ccdc.cam.ac.uk/structures/.

## Conflict of Interest

The authors declare no conflict of interest.

## Supporting information



Supporting Information

## Data Availability

The data that support the findings of this study are available in the supplementary material of this article.
