# Supplementary material for: Non‐Bonded Interaction Driven Morphology Evolution of CuMOF@MXene Energetic Composites: Synergistic Optimization of Thermal Stability and Combustion Performance
Source: Adv Sci (Weinh). 2025 Jul 13;12(38):e08755. doi: 10.1002/advs.202508755 (PMC12520471; doi:10.1002/advs.202508755)
Supplement: Supplementary file 1 — Supporting Information [file ADVS-12-e08755-s001.docx]

Supporting Information

Non-Bonded Interaction Driven Morphology Evolution of CuMOF@MXene Energetic Composites: Synergistic Optimization of Thermal Stability and Combustion Performance

Ke-Juan Meng, Xinwen Ma, Kunyu Xiong, Xiaoxia Ma, ^*^ Iftikhar Hussain, Momang Tian, Kaili Zhang ^*^

^*^ Corresponding author email: maxiaoxia@bit.edu.cn, [kaizhang@cityu.edu.hk](mailto:kaizhang@cityu.edu.hk) (Prof. Kaili Zhang)

**Contents**

**S1. Supporting Information Text**

**S2. Supplementary figures**

**S3. Supplementary tables**

**S1. Supporting Information Text**

**The DFT calculation method**

We performed all spin-polarized density-functional theory (DFT) calculations in the generalized gradient approximation (GGA) (1) using the Perdew-Burke-Ernzerhof (PBE) formulation with the Vienna Ab Initio Package (VASP). (2, 3) We chose the projected augmented wave (PAW) potentials (4, 5) to describe the ionic core and took valence electrons into account using a plane-wave basis set with a kinetic energy cut-off of 450 eV. Partial occupation of the Kohn-Sham orbitals was allowed using Gaussian smearing with a width of 0.05 eV. When the energy variation was less than 10-5 eV, the electron energies were considered to be self-consistent. Geometry optimization is considered to be convergent when the energy variation is less than 0.02 eV Å-1. The vacuum spacing in the direction perpendicular to the plane of the structure is 18 Å. The weak interactions are described using the DFT+D3 method with empirical corrections from the Grimme scheme. (6)

**The calculation method of flame area by FCM algorithm**

The idea of the FCM algorithm is that after initializing the cluster center of the image, the membership degree of each pixel to the cluster center and the new cluster center are solved according to the formula, to achieve the optimal target criterion function. (7) The objective function of FCM algorithm is:

$\begin{aligned} J\left( U,V \right)=\sum_{k=1}^{n} \sum_{i=1}^{c} u_{ik}^{m}d_{ik}^{2}\left( x_{k},v_{i} \right)\#\left( 1 \right) \end{aligned}$

In the Formula 1, $c$ represents the number of clusters, $u_{ik}(i=1,\ldots,c,k=1,\ldots,n)$ represents the membership degree of $x_{k}$ to the $i$th cluster,$u_{ik}\in\left[ 0,1 \right]$,$m$ represents the fuzzy coefficient, and $d_{ik}(x_{k},v_{i})$ represents the Euclidean distance of $x_{k}$ to the $i$th cluster:

$$\begin{aligned} d_{ik}\left( x_{k},v_{i} \right)=\left\| x_{k}-v_{i} \right\|\#\left( 2 \right) \end{aligned}$$

The Lagrange method is used to obtain:

$$\begin{aligned} u_{ik}=\left( \sum_{j=1}^{c} \left( \frac{d\left( x_{k},v_{i} \right)}{d\left( x_{k},v_{j} \right)} \right)^{\frac{2}{m-1}} \right)^{-1}\#\left( 3 \right) \end{aligned}$$

$$\begin{aligned} v_{i}=\frac{\sum_{k=1}^{n} \left( u_{ik} \right)^{m}x_{k}}{\sum_{k=1}^{n} \left( u_{ik} \right)^{m}}\#\left( 4 \right) \end{aligned}$$

The flow of the algorithm is: (1) initialize, $v_{i}$, $m$ and $m$. (2) Update the membership matrix according to Formula 3. (3) Update the clustering center according to Formula 4. (4) Iterate until $\left\| v_{i+1}-v_{i} \right\|<\varepsilon(\varepsilon>0)$, in which $\varepsilon$ is the threshold given in advance, and otherwise continue to perform the step. According to the actual image size of 8.17 cm × 8.17 cm, the maximum flame area can be obtained by FCM algorithm, as shown in Figure S5.

**S2. Supplementary figures**


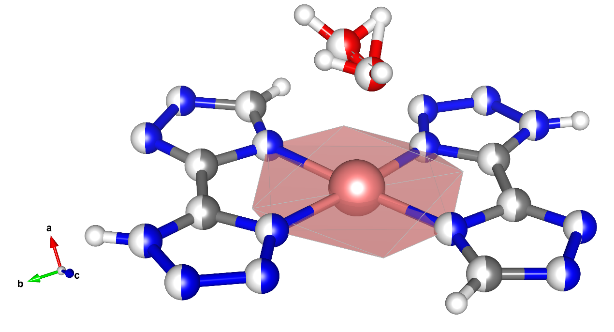


**Fig. S1.** The coordination environment of the Cu^2+^ ion.


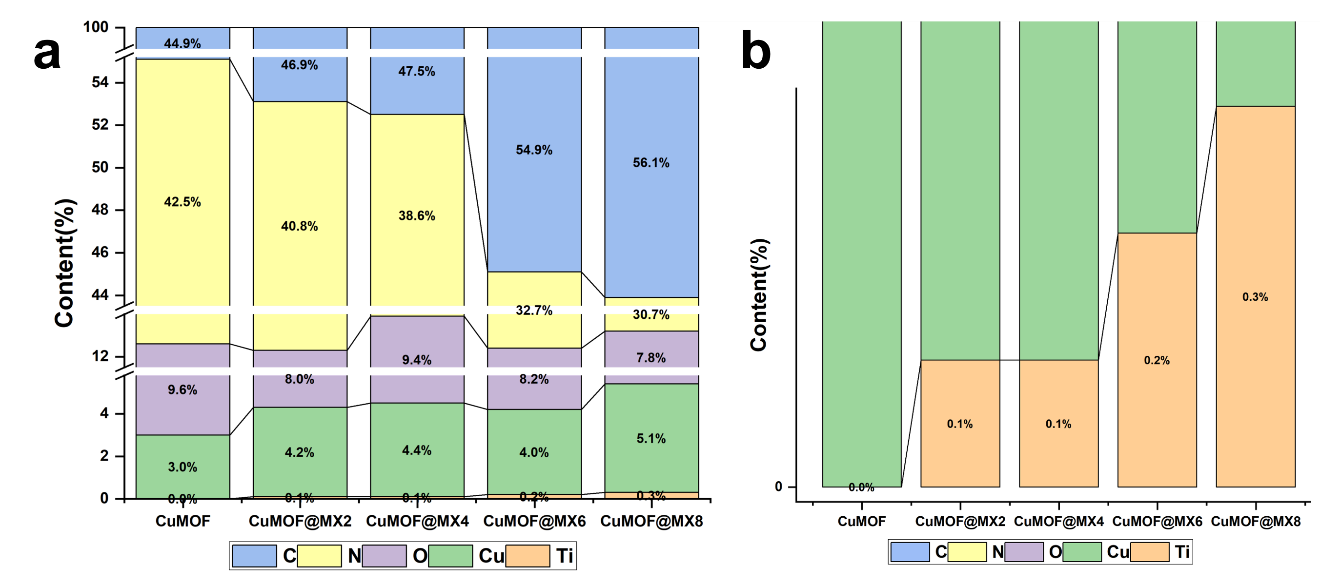


**Fig. S2.** EDS results of CuMOF and CuMOF@MX*_x_*.


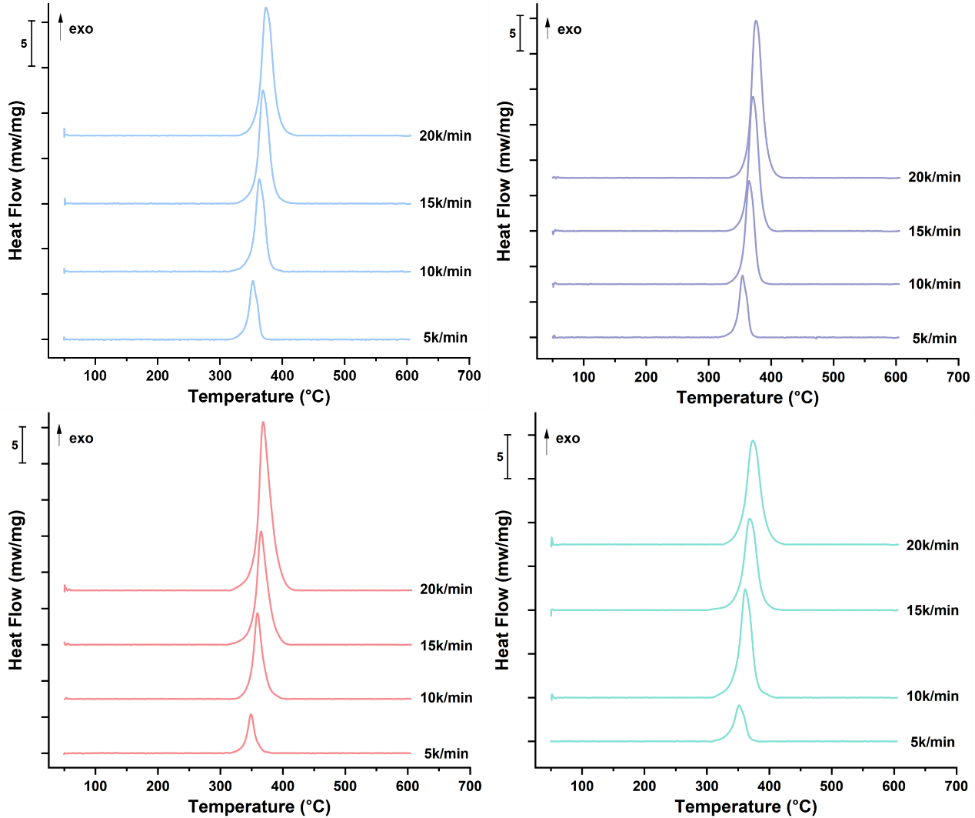


**Fig. S3.** DSC curves of CuMOF@MX_2_, CuMOF@MX_4_, CuMOF@MX_6_ and CuMOF@MX_8_ at different heating rates of 5, 10, 15, and 20 °C·min^−1^.


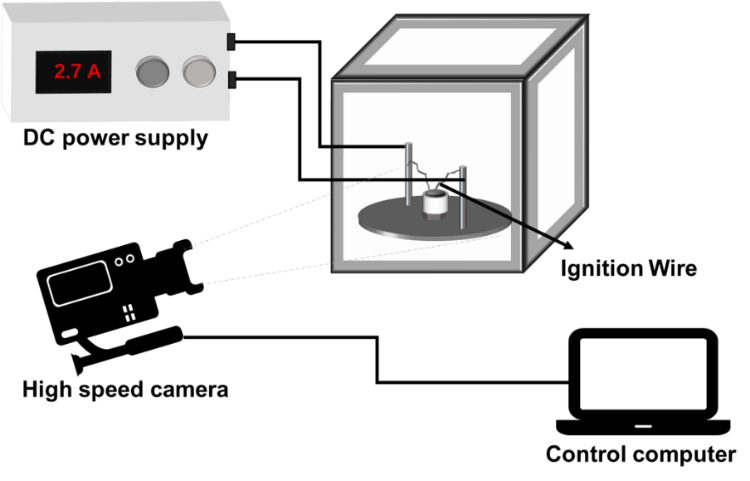


**Fig S4.** The equipment for open burning test.


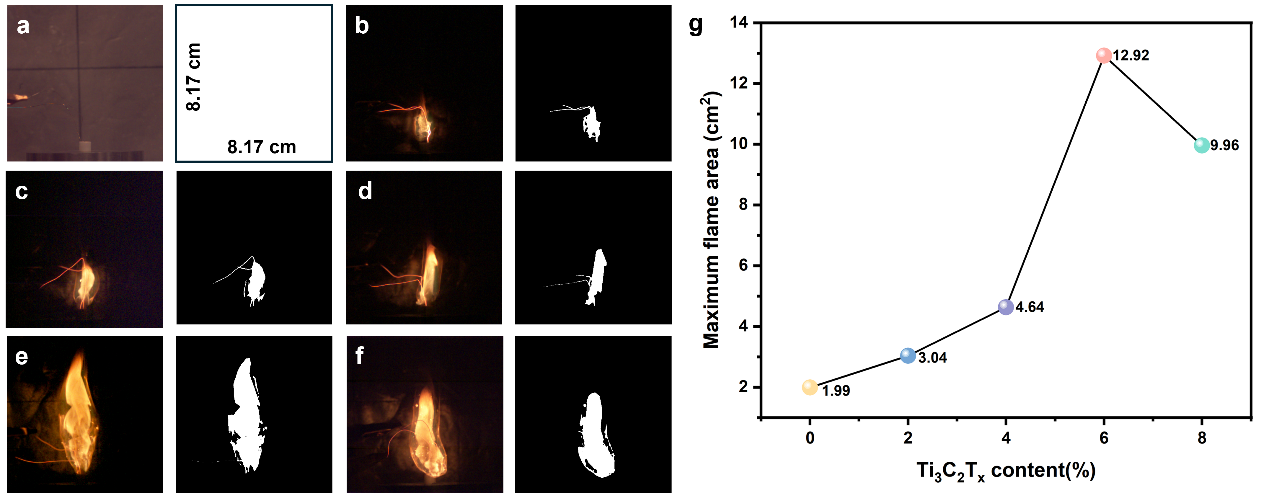


**Fig. S5.** (a) Actural image size of view. (b-f) The segmentation result of the maximum flame area of CuMOF and CuMOF@MXx. g) The maximum flame obtained by calculation.

**S3. Supplementary tables**

**Table S1.** Crystal data and structure refinement details for CuMOF.

|  | Cu(H_2_tztr)_2_(H_2_O)_2_ |
| --- | --- |
| CCDC number | 2425082 |
| Empirical formula | CuC6H8N14O2 |
| Formula weight | 371.81 |
| Temperature/K | 296 |
| Crystal system | Monoclinic/15 |
| Space group | C12/c1 |
| a/Å | 7.9282 |
| b/Å | 12.572 |
| c/Å | 13.390 |
| β/º | 98.731 |
| Volume/ Å^3^ | 1319.2 |
| Z | 4 |
| ρ_calc_ g/cm^3^ | 1.882 |
| µ/mm^-1^ | 1.701 |
| F(000) | 748 |
| Crystal size/mm^3^ | 0.09 0.11 0.13 |
| Radiation | MoKα(λ=0.71073) |
| 2Φrange for data collection/º | 3.06 to 30.42 |
| Index ranges | -11≤h≤11,-17≤k≤17,-12≤l≤19 |
| Reflections collections | 7315 |
| Independent reflections | 2021[R_in_=0.0625] |
| Data/restraints/parameters | 2021/152/205 |
| Goodness-of-fit on F^2^ | 1.113 |
| Final R indexes [all data] | R1=0.0625, wR2=0.1350 |
| Largest diff. peak/hole/e Å^-3^ | 0.391/-0.523 |

**Table S2.** DSC parameters of CuMOF and CuMOF@MX*_x_* hybrids at a heating rate of 10 °C·min^−1^.

| Samples | Exothermic peak | | | |
| --- | --- | --- | --- | --- |
|  | *T*_i_ /ºC | *T*_p_ /ºC | *T*_e_/ºC | *Δ*H/J·g^-1^ |
| CuMOF | 314.9 | 338.1(T_p1_), 365.3(T_p2_) | 377.9 | 1228 |
| CuMOF@MX_2_ | 350.8 | 366.1 | 379.4 | 1384 |
| CuMOF@MX_4_ | 354.2 | 364.7 | 380.4 | 1462 |
| CuMOF@MX_6_ | 347.1 | 359.5 | 375.1 | 1319 |
| CuMOF@MX_8_ | 346.3 | 361.8 | 379.8 | 1295 |

a), *T*_i_, the initial temperature of runaway reaction; *T*_p_, peak temperature of exothermic process; *T*_e_, the end temperature for heat change; *Δ*H, heat release.

**Table S3.** Non-isothermal DSC curves for the decomposition of CuMOF and CuMOF@MX*_x_* hybrids at different heating rates.

| Samples | β/ºC·min^-1^ | Exothermic peak | | | |
| --- | --- | --- | --- | --- | --- |
|  |  | *T*_h_ /mW/mg | *T*_p_ / °C | *T*_w_ / °C | symmetry |
| CuMOF | 5 | 1.71 | 328.9 | 30 | 0.15 |
|  | 10 | 3.17 | 338.1 | 37.5 | -0.16 |
|  | 15 | 3.06 | 344.6 | 36.8 | 0.09 |
|  | 20 | 3.75 | 349.4 | 37.9 | 0.16 |
| CuMOF@MX_2_ | 5 | 6.06 | 354.2 | 15.86 | -0.19 |
|  | 10 | 9.69 | 366.1 | 18.1 | -0.12 |
|  | 15 | 11.01 | 370.6 | 19.3 | 0.01 |
|  | 20 | 13.72 | 375.5 | 21.25 | 0.04 |
| CuMOF@MX_4_ | 5 | 8.2 | 355.3 | 14.89 | -0.11 |
|  | 10 | 13.9 | 364.7 | 16.1 | -0.09 |
|  | 15 | 18.36 | 371.8 | 17.3 | 0.008 |
|  | 20 | 21.48 | 376.6 | 18.7 | 0.08 |
| CuMOF@MX_6_ | 5 | 4.72 | 349.1 | 14.5 | 0.01 |
|  | 10 | 10.4 | 359.5 | 17.1 | 0.11 |
|  | 15 | 13.9 | 366.04 | 20.2 | 0.14 |
|  | 20 | 20.9 | 369.7 | 21 | 0.21 |
| CuMOF@MX_8_ | 5 | 3.94 | 353.3 | 20.88 | -0.28 |
|  | 10 | 11.78 | 361.8 | 22.58 | -0.21 |
|  | 15 | 10.04 | 370.1 | 23.64 | -0.08 |
|  | 20 | 11.55 | 374.7 | 26.5 | -0.02 |

a), β, heating rate; *T*_h_, peak height of exothermic process; *T*_w_, peak width of exothermic process.

**SI References**

1. J. P. Perdew, K. Burke, M. Ernzerhof, Generalized gradient approximation made simple. *Phys Rev Lett* **77**, 3865-3868 (1996).

2. G. Kresse, J. Furthmuller, Efficiency of ab-initio total energy calculations for metals and semiconductors using a plane-wave basis set. *Computational Materials Science* **6**, 15-50 (1996).

3. G. Kresse, J. Furthmuller, Efficient iterative schemes for ab initio total-energy calculations using a plane-wave basis set. *Phys Rev B* **54**, 11169-11186 (1996).

4. G. Kresse, D. Joubert, From ultrasoft pseudopotentials to the projector augmented-wave method. *Phys Rev B* **59**, 1758-1775 (1999).

5. P. E. Blochl, Projector Augmented-Wave Method. *Phys Rev B* **50**, 17953-17979 (1994).

6. S. Grimme, J. Antony, S. Ehrlich, H. Krieg, A consistent and accurate ab initio parametrization of density functional dispersion correction (DFT-D) for the 94 elements H-Pu. *J Chem Phys* **132** (2010).

7. J. C. Bezdek, R. Ehrlich, W. Full, Fcm - the Fuzzy C-Means Clustering-Algorithm. *Comput Geosci* **10**, 191-203 (1984).
